# Supplementary material for: Complexome profiling of the Chlamydomonas psb28 mutant reveals TEF5 as an early PSII assembly factor
Source: Plant Cell. 2025 Mar 17;37(6):koaf055. doi: 10.1093/plcell/koaf055 (PMC12164590; doi:10.1093/plcell/koaf055)
Supplement: koaf055_Supplementary_Data [file koaf055_supplementary_data.zip › psb28_interactive.zip!/plots/intensity/qualityPlots/peptides/PSAH_Cre07.g330250.t1.2.html]

### Cre07.g330250.t1.2

Trivial name: PSAH  
SeqCoverage [median]: 73.80  
Mass: 14.15 kDa  
  
 

| Sequence | MissedCleavage | Mass [Da] | wtIntensity | muIntensity | Score |
| --- | --- | --- | --- | --- | --- |
| ALVALSGIAAIVTYGLK | 0 | 1659.00 | 90611.68 | 10761.98 | 231.66 |
| ALVALSGIAAIVTYGLKGAK | 1 | 1915.15 | 6771.62 | 6335.76 | 110.77 |
| DADLPITK | 0 | 871.47 | 1225780.55 | 634626.20 | 236.09 |
| DADLPITKGPQTTGENGK | 1 | 1840.92 | 6452.22 | 5534.56 | 73.21 |
| ESLRALVALSGIAAIVTYGLK | 1 | 2144.26 | 709.25 | 0.00 | 127.50 |
| FFTQATDIISR | 0 | 1297.67 | 888065.83 | 1305152.81 | 280.49 |
| FFTQATDIISRR | 1 | 1453.77 | 358738.50 | 332198.42 | 177.83 |
| GAKDADLPITK | 1 | 1127.62 | 96701.91 | 56415.44 | 145.09 |
| GPQTTGENGKGGSVR | 1 | 1443.71 | 6000.26 | 5278.23 | 143.38 |
| KRYPDNQAK | 2 | 1118.58 | 48621.52 | 41329.66 | 140.75 |
| RYPDNQAK | 1 | 990.49 | 25200.42 | 21216.74 | 164.77 |
| YFDLQDMENTTGSWDMYGVDEK | 0 | 2643.08 | 27911.07 | 12820.28 | 110.75 |
| YFDLQDMENTTGSWDMYGVDEKK | 1 | 2771.17 | 3084.75 | 0.00 | 51.03 |
| YGENSR | 0 | 724.31 | 764.09 | 2997.61 | 104.94 |
| YPDNQAK | 0 | 834.39 | 105642.10 | 103121.09 | 152.39 |
|  |  |  | 2891055.8 | 2537788.8 |  |
